# *Helicobacter pylori* infection and its impact on metabolic dysfunction-associated steatotic liver disease: a mediation analysis of neutrophil-albumin ratio

**DOI:** 10.3389/fnut.2025.1701544

**Published:** 2025-12-11

**Authors:** Liuying He, Haiyan Yang, Ziqin Zeng, Qian Wang, Haitao Guan, Ping Zhao

**Affiliations:** 1 Department of Gastroenterology, The Second Affiliated Hospital of Xi’an Jiaotong University, Xi’an, China; 2 Department of Health Management, The Second Affiliated Hospital of Xi’an Jiaotong University, Xi’an, China; 3 Department of Surgical Oncology, The Second Affiliated Hospital of Xi’an Jiaotong University, Xi’an, China

**Keywords:** MASLD, *Helicobacter pylori* infection, NAR, mediation analysis, cross-sectional study

## Abstract

**Background:**

*Helicobacter pylori*, a widely found bacterium, has been controversially connected to the risk of metabolic dysfunction-associated steatotic liver disease (MASLD). How the neutrophil–albumin ratio (NAR) influences the relationship between *H. pylori* infection and MASLD is unknown. Therefore, in this study, how *H. pylori* infection, the NAR, and MASLD are connected, including the possible impact of the NAR on the relationship between *H. pylori* and MASLD, was investigated.

**Methods:**

In this cross-sectional study, data from 26,245 medical check-ups conducted between January 2021 and August 2023 at a tertiary hospital located in northwestern China were used. *H. pylori* infection was used as the independent variable, with metabolic dysfunction-associated steatotic liver disease (MASLD) as the dependent variable and the neutrophil–albumin ratio (NAR) as a mediator. The associations between *H. pylori* infection, the NAR, and the risk of MASLD were evaluated with a logistic regression model, and mediation analysis confirmed the role of the NAR as a mediator.

**Results:**

Among 26,245 participants, the frequencies of *H. pylori* infection and MASLD were 30.5 and 25.8%, respectively, and the mean value of NAR was 0.72 ± 0.241. The analysis using multiple logistic regression indicated a link between *H. pylori* infection and NAR (Q2: OR = 1.293, 95% CI: 1.199–1.396; Q3: OR = 1.364, 95% CI: 1.263–1.472; Q4: OR = 1.517, 95% CI: 1.406–1.638) and MASLD (OR = 1.226, 95% CI: 1.156–1.301). RCS analysis revealed a significant positive non-linear relationship. The mediation effect analysis found that *H. pylori* directly contributed to MASLD development (*β* = 0.014, *p* = 0.004), and NAR partially mediated the indirect effect of *H. pylori* on MASLD (*β* = 0.008, *p* < 0.001), with 35.77% of the effect being mediated.

**Conclusion:**

There was a positive correlation between *H. pylori* infection and MASLD risk, with NAR partially mediating this connection. This study provides clinical evidence elucidating the impact of *Helicobacter pylori* infection on MASLD.

## 1 Introduction

Metabolic dysfunction-associated steatotic liver disease (MASLD) is characterized by an unusual buildup of fat in liver cells and is frequently associated with obesity, type 2 diabetes, and metabolic problems. MASLD encompasses a range of disorders that include simple fatty liver, metabolic-associated steatohepatitis (MASH), liver fibrosis, and even hepatocellular carcinoma (HCC) (1, 2). In 43–44% of cases, MASLD progress to MASH, and 7–30% of MASLD patients develop liver scarring or cirrhosis (3). Thus, investigating the risk factors and pathogenesis of MASLD is crucial for its prevention and treatment.

The neutrophil–albumin ratio (NAR) combines data on chronic inflammation and metabolic conditions and plays a crucial role in clinical settings. A team of researchers reported that the NAR demonstrates the best predictive performance for the risk of MASLD, with an AUC value of 0.813 (4). In addition, compared with the NLR and the SII, the NAR demonstrates a more significant predictive value for all-cause and cardiovascular disease (CVD) mortality in patients with MASLD (5).

*Helicobacter pylori* is a gram-negative bacterium found in the gastric mucosa and is widely recognized for its carcinogenic properties. Globally, *H. pylori* infection is widespread; China is a region with a high prevalence of *H. pylori* infection, affecting approximately 50% of its citizens. Moreover, provinces show significant variation in infection rates, with adults experiencing rates ranging from 24.3 to 69.3% and children experiencing rates ranging from 2.9 to 46.3% (6). Previous studies have proposed that MASLD may arise from continuous inflammation caused by *H. pylori* infection (7, 8), insulin resistance (9), and lipid metabolism disorders (9–11). Several observational studies and meta-analyses have provided evidence that *H. pylori* infection could be linked to a higher risk of MASLD, although the findings are not consistent (11–15).

An investigation involving 3,509 individuals also revealed that *H. pylori* infection is linked to higher neutrophil counts and a higher systemic inflammatory response index (SIRI) (16), and another study involving 6,349 participants revealed a significant association between *H. pylori* infection and reduced serum albumin levels (17). These data indicate a possible association between *H. pylori* infection and the NAR. Nevertheless, extensive research on the interaction between *H. pylori* infection and the NAR and the relationship among *H. pylori* infection, the NAR, and MASLD risk is lacking.

The objective of this research was to investigate the potential association between *H. pylori* infection, the NAR, and MASLD by analysing data from a cross-sectional study conducted on a cohort of patients who underwent physical examination at a tertiary hospital in northwestern China from January 2021 to August 2023 and to investigate the potential link between *H. pylori* infection, the NAR, and MASLD.

## 2 Methods

### 2.1 Research design and target population

**Figure 1:**
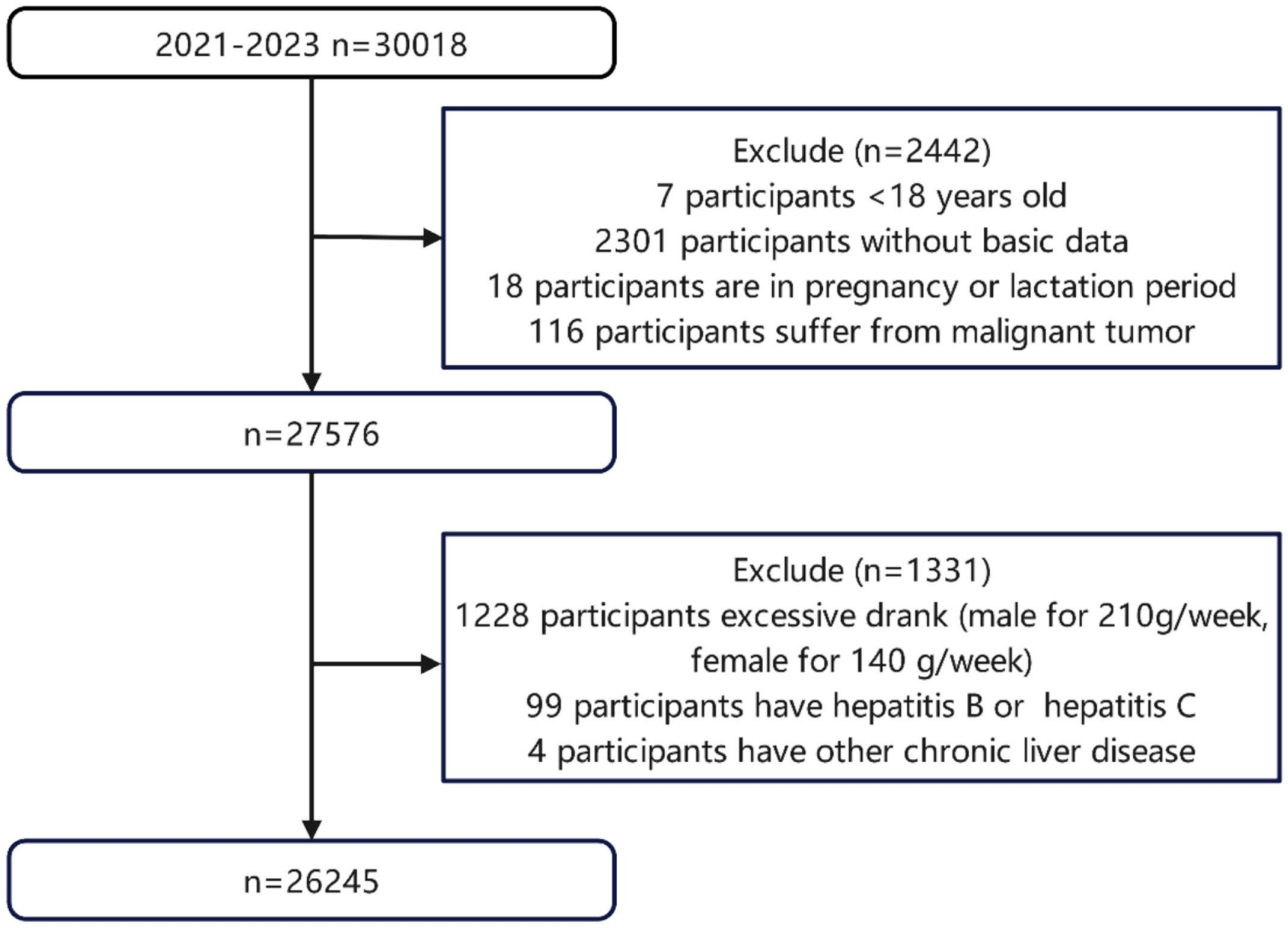
Flowchart of study participants.

In this cross-sectional study, individuals who underwent extensive physical examinations at a tertiary hospital in northwestern China between January 2021 and August 2023 were assessed. Participants who underwent abdominal ultrasound (US) and 13C urea breath testing (13C-UBT) and completed a questionnaire were included in the study. Individuals were excluded from the study if they met any of the following criteria: under the age of 18, insufficient basic data, pregnancy or lactation, a prior history of malignancy, excessive alcohol consumption (defined as more than 210 grams per week for males and 140 grams per week for females), positive test results for hepatitis B or C viruses, or a history of chronic liver conditions, including drug-induced liver injury, autoimmune hepatitis, hepatomegaly, or cirrhosis. The analysis included 26,245 participants in total (Figure 1). The study was approved by the Medical Ethics Committee of the Second Affiliated Hospital of Xi’an Jiaotong University (Trial Registration Number: 2025077). The STROBE guidelines for presenting data from observational studies were followed (18).

### 2.2 Assessment of MASLD

We used abdominal ultrasound to assess whether the participants had fatty liver disease. Serum was collected from participants and tested for triglyceride (TG), total cholesterol, low-density lipoprotein (LDL-C), high-density lipoprotein (HDL-C), and fasting glucose (FPG) levels. Participants’ height, weight, waist circumference, hip circumference, and systolic and diastolic blood pressure were determined by physical examination, and BMI was calculated in kg/m2. A questionnaire was used to assess the participants’ history of hypertension, diabetes, and related medications. The Asian Pacific Association for the Study of the Liver Clinical Practice Guidelines for the Diagnosis and Management of Metabolic-Associated Fatty Liver Disease (19) were used to diagnose MASLD.

### 2.3 Assessment of NAR

We used the neutrophil-albumin ratio (NAR) as a continuous variable and obtained the neutrophil count and serum albumin data at baseline. The NAR calculated performed using the following formula (4): NAR = neutrophil count (1,000 cells per microlitre) divided by albumin (grams per decilitre).

### 2.4 Assessment of *Helicobacter pylori* infection

Participants must be off PPIs, H₂ -blockers, antibiotics, bismuth, and endoscopy for ≥4 weeks before *H. pylori* retesting with ^13^C-urea test (UBT). All assays were performed by a single technician using a calibrated isotope-ratio mass spectrometer to ensure reproducibility and quality control.

### 2.5 Covariates

Data on covariates such as sex, age, education, marital status, occupation, smoking and drinking status, neutrophil count, and serum albumin concentration were collected. Smoking status was classified as nonsmoker, ex-smoker, or current smoker on the basis of the following criteria: Participants who had a lifetime cigarette consumption of fewer than 100 cigarettes were identified as nonsmokers; persons who had smoked more than 100 cigarettes during their lifetime but were not presently smoking were designated as ex-smokers; and those who had consumed more than 100 cigarettes in their lifetime and had not ceased smoking were labelled as current smokers. A history of drinking was measured by weekly alcohol intake according to the China Chronic Disease and its Risk Factor Detection Report 2010 (20).

### 2.6 Statistical analysis

The data were tested and found to conform to a normal distribution. Multiple covariance analysis revealed no covariance (VIF < 5). Continuous variables are presented as the mean and standard deviation, whereas categorical variables are presented as the frequency and percentage. A t test was used to analyse normally distributed data, and a chi-square test was applied to categorical data to examine differences between individuals with and without *H. pylori* infection.

In this study, three analytical models were developed using univariate and multivariate logistic regression to explore the connection between *H. pylori* infection, the NAR, and MASLD: an unadjusted model (Model 1), a minimally adjusted model (Model 2, including age and sex) and a fully adjusted model (Model 3, considering age, sex, educational background, marital status, job status, smoking behavior, and drinking habits). The 95% confidence interval (CI) of the odds ratio (OR) was used to determine the effect size. We converted the NAR from a continuous variable into a categorical variable by splitting the participants into quartiles and computed trend *p* values to ensure that the results were consistent across these variable types. We further explored the nonlinear relationship between the NAR and MASLD through RCS analysis.

In the subgroup analyses, the statistical methods previously outlined were utilized, and we carried out interaction tests to assess potential variations in the relationship between *H. pylori* infection and MASLD risk on the basis of sex, age, smoking status, and alcohol consumption.

A mediation analysis was conducted to determine how the NAR mediates the connection between *H. pylori* infection and MASLD risk, with corrections for variables such as age, sex, education, marital status, occupation, smoking status, and alcohol consumption status.

Empower Stats Software and R language were used for statistical analyses. Two-tailed *p* values less than 0.05 were considered to indicate statistical significance.

## 3 Results

### 3.1 Participant’s baseline characteristics

Primary baseline characteristics, stratified by the *H. pylori* infection status of the participants, are presented in Table 1. Among the 26,245 individuals in the study, the average age was 38.81 ± 11.155 years, and 45.9% of the participants were female. The percentages of participants with HP infection and MASLD were 30.5 and 25.8%, respectively. The mean neutrophil–albumin ratio (NAR) was 0.72 ± 0.241. On the basis of the C13 breath test results, participants were divided into two categories: those without *H. pylori* infection (*n* = 18,244) and those with *H. pylori* infection (*n* = 8,001). Compared with those without *H. pylori* infection, participants with *H. pylori* infection were more likely to be older, male, married, have lower education levels, work in manual jobs, and have a history of smoking or drinking. In comparison to the group without *H. pylori* infection, the infected group demonstrated significantly elevated neutrophil counts and NARs, along with a marked decrease in serum albumin levels (*p* < 0.001).

**Table 1:** Baseline features of participants with different *H. pylori* infection statuses.

Variables	Total	*H. pylori*− (*n* = 18,244)	*H. pylori* + (*n* = 8,001)	*p* value
Age, years	38.81 ± 11.155	38.57 ± 11.112	39.36 ± 11.235	<0.001
Gender (*n* %)				<0.001
Male	14,216 (54.167%)	9,650 (52.894%)	4,566 (57.068%)	
Female	12,028 (45.882%)	8,594 (47.106%)	3,435 (42.932%)	
Marriage (*n* %)				<0.001
Unmarried	5,619 (21.410%)	4,068 (22.298%)	1,551 (19.385%)	
Married	20,068 (76.464%)	13,792 (75.598%)	6,276 (78.440%)	
Others	558 (2.126%)	384 (2.105%)	174 (2.175%)	
Education level (*n* %)				<0.001
Below junior high school	1,370 (5.220%)	831 (4.555%)	539 (6.737%)	
Senior high school	2,302 (8.771%)	1,492 (8.178%)	810 (10.124%)	
Bachelor degree	17,997 (68.573%)	12,594 (69.031%)	5,403 (67.529%)	
Postgraduate degree	4,576 (17.436%)	3,327 (18.236%)	1,249 (15.611%)	
Occupation (*n* %)				<0.001
Nonphysical laborers	23,688 (90.257%)	16,565 (90.797%)	7,123 (89.026%)	
Physical laborers	2,557 (9.743%)	1,679 (9.203%)	878 (10.974%)	
Smoking status (*n* %)				<0.001
Never	19,059 (72.620%)	13,508 (74.041%)	5,551 (69.379%)	
Former	1,627 (6.199%)	1,074 (5.887%)	553 (6.912%)	
Current smoker	5,559 (21.181%)	3,662 (20.072%)	1897 (23.710%)	
Drinking status (*n* %)				0.025
Never	14,251 (54.300%)	9,990 (54.758%)	4,261 (53.256%)	
Moderate	11,994 (45.700%)	8,254 (45.242%)	3,740 (46.744%)	
BMI	23.82 ± 3.608	23.70 ± 3.498	24.10 ± 3.832	<0.001
SBP (mmHg)	121.00 ± 14.278	120.67 ± 14.111	121.74 ± 14.623	<0.001
DBP (mmHg)	76.84 ± 10.431	76.59 ± 10.292	77.40 ± 10.719	<0.001
Waistline	81.24 ± 10.784	80.84 ± 10.738	82.14 ± 10.888	<0.001
Triglycerides (mmol/L)	1.51 ± 1.220	1.48 ± 1.171	1.57 ± 1.323	<0.001
HDL-C (mmol/L)	1.32 ± 0.315	1.33 ± 0.314	1.29 ± 0.317	<0.001
LDL-C (mmol/L)	2.67 ± 0.741	2.67 ± 0.741	2.69 ± 0.739	0.005
FBG (mmol/L)	5.17 ± 0.986	5.14 ± 0.929	5.21 ± 1.105	<0.001
Neutrophils (10^9/L)	3.34 ± 1.107	3.30 ± 1.110	3.44 ± 1.095	<0.001
Albumin (g/L)	46.85 ± 2.603	46.94 ± 2.598	46.64 ± 2.602	<0.001
NAR	0.72 ± 0.241	0.71 ± 0.241	0.74 ± 0.239	<0.001
History of hypertension (*n* %)	1843 (7.022%)	1,197 (6.811%)	646 (8.368%)	<0.001
History of diabetes (*n* %)	562 (2.141%)	359 (2.043%)	203 (2.630%)	0.004
MASLD (*n* %)	6,761 (25.761%)	4,479 (24.551%)	2,282 (28.521%)	<0.001

*H. pylori*, *Helicobacter pylori*; BMI, body mass index; SBP, Systolic blood pressure; DBP, Diastolic blood pressure; HDL-C, High-density lipoprotein-cholesterol; LDL-C, Low-density lipoprotein-cholesterol; FBG, Fasting blood glucose; NAR, Neutrophil-Albumin Ratio.

### 3.2 Association between *Helicobacter pylori* and NAR

**Figure 2:**
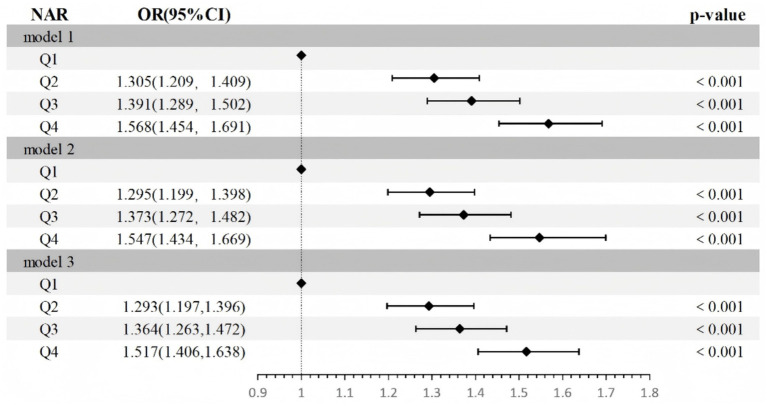
Relationship of NAR with *H. pylori* infection. Model 1: Unadjusted, Model 2: Adjusted for age and sex, Model 3: Adjusted for age, sex, occupation, education, Marriage, smoking status, drinking status. CI, confidence interval; NAR, neutrophil-albumin ratio.

We transformed the NAR into a categorical variable using the quartile method and incorporated it into a logistic regression model (Figure 2). A positive association between *H. pylori* infection and the NAR was revealed by the fully adjusted model results (Q2: OR = 1.293, 95% CI: 1.199–1.396, *p* < 0.001; Q3: OR = 1.364, 95% CI: 1.263–1.472, *p* < 0.001; Q4: OR = 1.517, 95% CI: 1.406–1.638, *p* < 0.001).

### 3.3 Association between NAR and MASLD

Additionally, we developed three logistic regression models to investigate the link between the NAR and MASLD (Table 2). According to the unadjusted model, each 1-unit increase in the NAR was associated with a 4.713-fold greater likelihood of developing MASLD (OR = 4.713; 95% CI: 4.205–5.282; *p* < 0.001). After partial adjustment for age and sex, the estimate remained essentially unchanged (OR = 4.588; 95% CI: 4.065–5.180; *p* < 0.001). According to the fully adjusted model (Model 3), each 1-unit increase in the NAR resulted in a 4.443-fold increase in the risk of MASLD (OR = 4.443; 95% CI: 3.927–5.026; *p* < 0.001). As confounders were progressively adjusted for, NAR remained a risk factor for MASLD.

**Table 2:** Relationship of *H. pylori* infection and NAR with MASLD.

Characteristic	Model 1		Model 2		Model 3	
	OR (95%CI)	*p*-value	OR (95%CI)	*p*-value	OR (95%CI)	*p*-value
*H. pylori* negative	Ref		Ref		Ref	Ref
*H. pylori* positive	1.226 (1.156, 1.301)	<0.001	1.160 (1.090, 1.234)	<0.001	1.141 (1.072, 1.215)	<0.001
NAR continuous	4.713 (4.205, 5.282)	<0.001	4.588 (4.065, 5.180)	<0.001	4.443 (3.927, 5.026)	<0.001
NAR Q1	Ref		Ref		Ref	Ref
NAR Q2	1.673 (1.530, 1.828)	<0.001	1.567 (1.429, 1.718)	<0.001	1.564 (1.425, 1.716)	<0.001
NAR Q3	2.379 (2.183, 2.593)	<0.001	2.171 (1.985, 2.374)	<0.001	2.144 (1.959, 2.345)	<0.001
NAR Q4	3.119 (2.867, 3.394)	<0.001	2.918 (2.672, 3.187)	<0.001	2.868 (2.623, 3.136)	<0.001
P for trend	<0.001		<0.001		<0.001	

Model 1: Unadjusted.

Model 2: Adjusted for age and sex.

Model 3: Adjusted for age, sex, occupation, education, Marriage, smoking status, drinking status.

CI, confidence interval; OR, odds ratio; NAR, neutrophil-albumin ratio.

A sensitivity analysis was carried out to test the robustness of the findings. By employing the quartile method, the NAR was converted into a categorical variable and added back into the logistic regression model to evaluate the trend. The fully adjusted model demonstrated that MASLD risk increased with increasing NAR (Q2: OR = 1.564, 95% CI: 1.425–1.716, *p* < 0.001; Q3: OR = 2.144, 95% CI: 1.959–2.345, *p* < 0.001; Q4: OR = 2.868, 95% CI: 2.623–3.136, *p* < 0.001). The findings from the trend test (P for trend < 0.001) demonstrated that when treated as a categorical variable, the NAR was consistent with the continuous variable results (Table 2).

**Figure 3:**
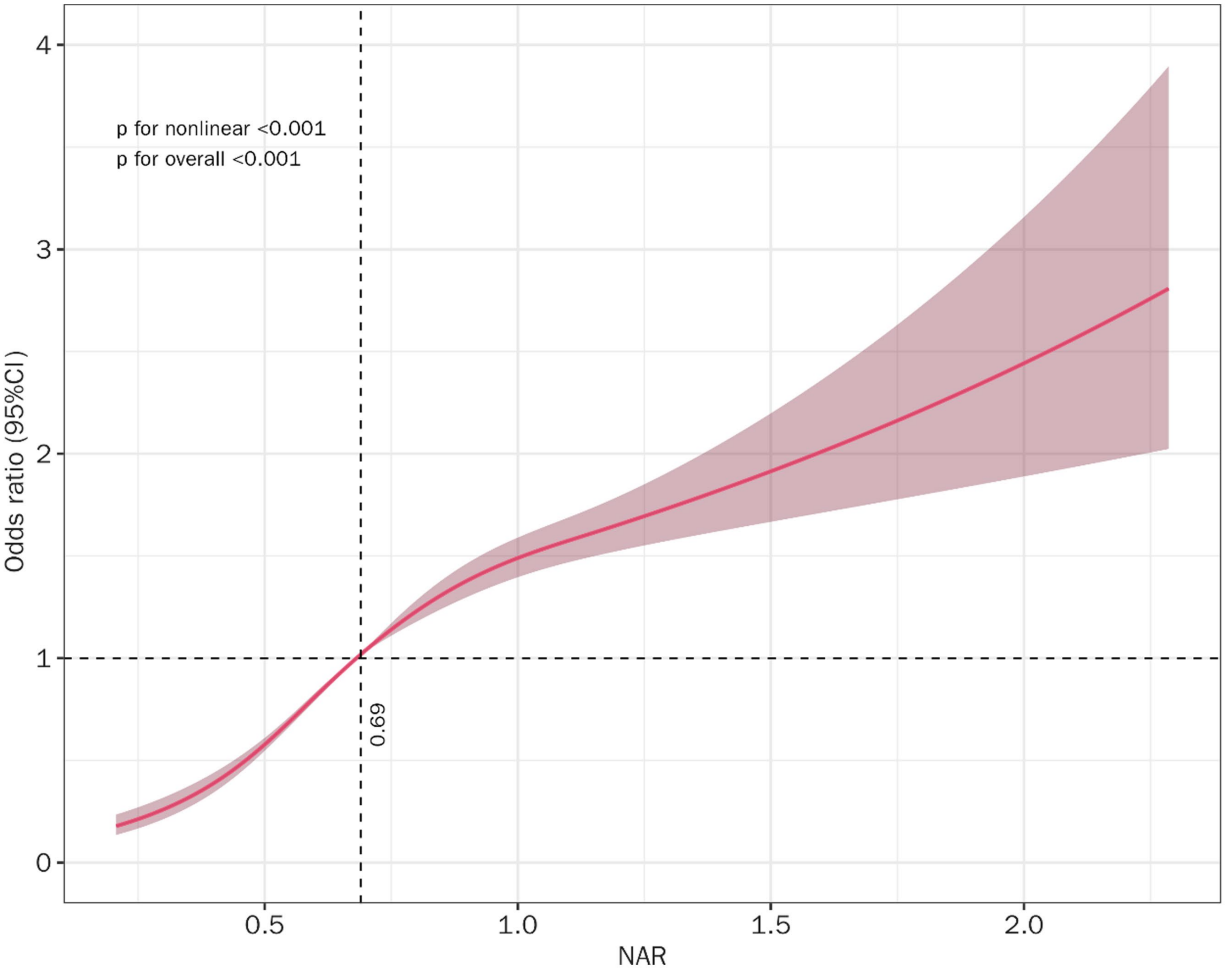
Nonlinear connection of neutrophil-albumin ratio (NAR) with MASLD. Adjusted for age, sex, occupation, education, Marriage, smoking status, drinking status.

An RCS analysis was carried out to provide more insight into the connection between NAR and MASLD risk. Across unadjusted, partially adjusted (Supplementary Figures S1, S2) and fully adjusted models (Figure 3), a robust nonlinear positive correlation between NAR and MASLD risk persisted (p for nonlinearity < 0.001).

### 3.4 Associations between *Helicobacter pylori* infection and MASLD

A logistic regression model was used to assess the connection between *H. pylori* infection and MASLD. Unadjusted analysis indicated that people with *H. pylori* infection had a 22.6% greater likelihood of developing MASLD (OR = 1.226, 95% CI: 1.156–1.301; *p* < 0.001). The partially adjusted model, controlling for age and sex, indicated that infected individuals had a 16.0% increased likelihood of developing MASLD (OR = 1.160, 95% CI: 1.090–1.234; *p* < 0.001). The risk of MASLD was 14.1% greater for *H. pylori*-infected individuals, as shown by the fully adjusted model (OR = 1.141, 95% CI: 1.072–1.215; *p* < 0.001) (Table 2).

**Figure 4:**
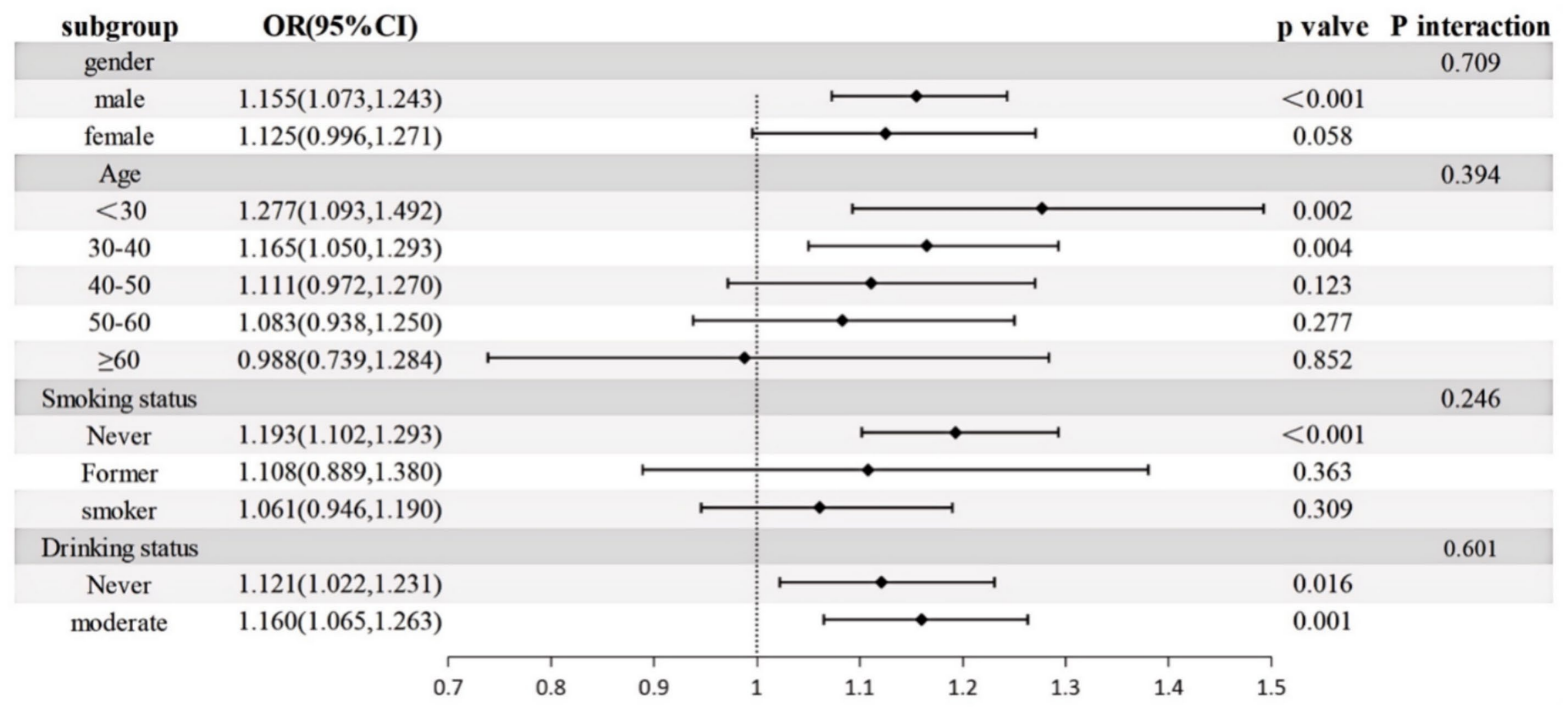
Subgroup analysis of MASLD. Adjusted for age, sex, occupation, education, Marriage, smoking status, drinking status.

The connection between *H. pylori* infection and MASLD risk was examined through subgroup analyses, considering age, sex, smoking status, and alcohol consumption, and an interaction test was performed, which revealed (P for interaction > 0.05) that there was no significant difference in the results among the subgroups (Figure 4).

### 3.5 Mediation effect of the NAR on the association between *Helicobacter pylori* and MASLD

**Figure 5:**
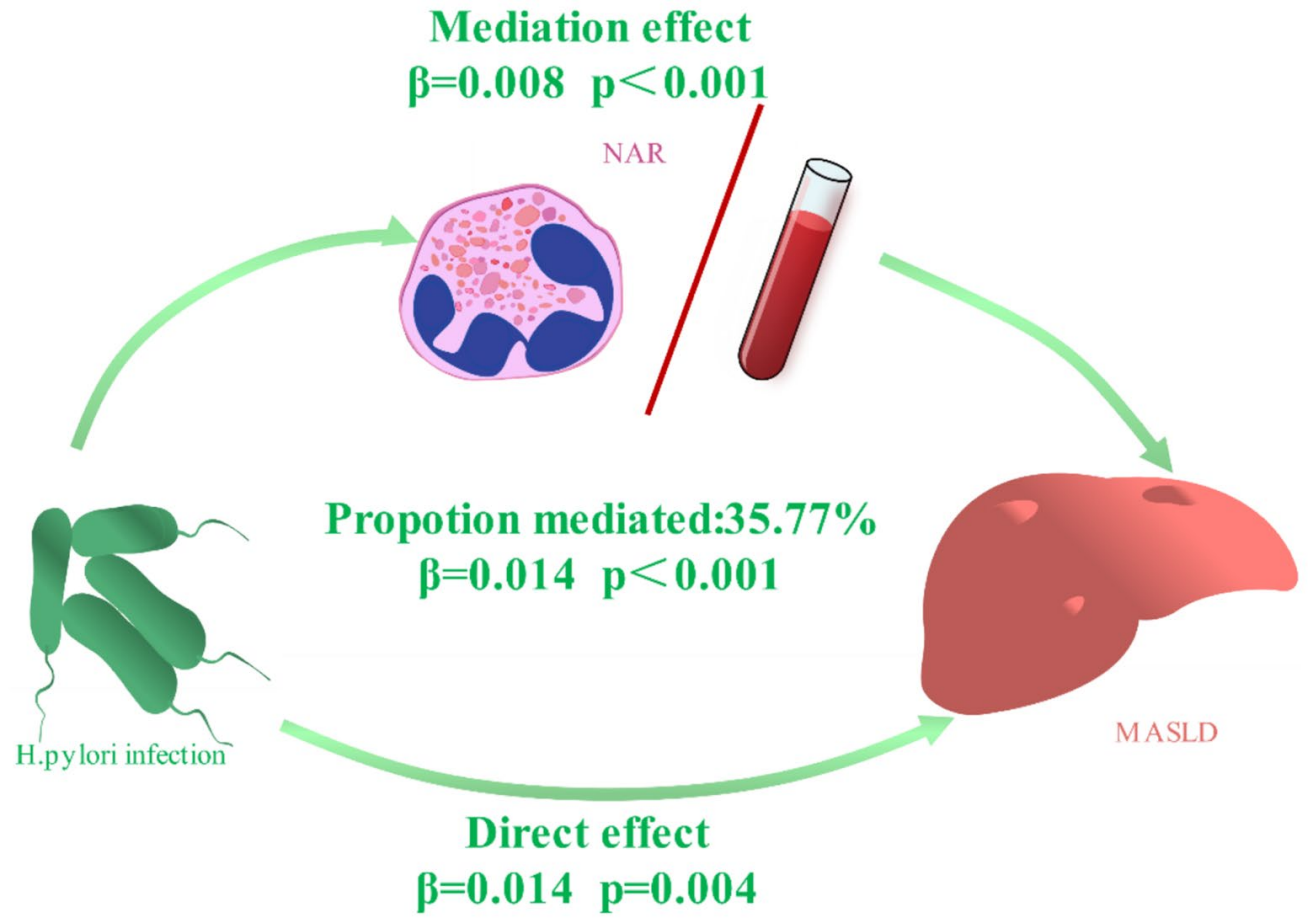
Mediation effects of the NAR between *H. pylori* infection and MASLD. Adjusted for age, sex, occupation, education, Marriage, smoking status, drinking status. Figure elements were sourced and adapted from Chilton. J via SciDraw.io, licensed under CC BY 4.0.

These results suggest that the NAR is involved in the mechanism that connects *H. pylori* infection with MASLD. Our study focused on the mediating effect of the NAR on the connection between *H. pylori* infection and MASLD, and we used mediation effect analysis to explore the inherent relationship (Figure 5). The results from the fully adjusted model revealed that *H. pylori* infection directly affected the risk of MASLD (*β* = 0.014, *p* = 0.004), with the NAR serving as a partial mediator for its indirect impact on MASLD risk (*β* = 0.008, *p* < 0.001), contributing to 35.77% of the effect.

## 4 Discussion

This cross-sectional study investigated the relationships between *H. pylori* infection and both the NAR and MASLD and investigated whether the NAR mediates the effect of *H. pylori* infection on MASLD by analysing the physical examination records of 26,245 participants at a tertiary hospital in northwest China. This study revealed a positive correlation between *H. pylori* infection and both the NAR and MASLD and a positive correlation between the NAR and MASLD. The analysis of mediation effects revealed that *H. pylori* infection directly influenced the development of MASLD (*β* = 0.014, *p* = 0.004), whereas the NAR partially mediated the indirect effect of *H. pylori* infection on MASLD risk (*β* = 0.008, *p* < 0.001), with a mediation effect ratio of 35.77%. These findings did not differ across sensitivity and stratification analyses.

We found a positive correlation between *H. pylori* infection and the NAR (Figure 2). These findings suggest that *H. pylori* infection may lead to persistent mild inflammation and nutrient-related metabolic issues. Numerous proinflammatory factors (IL-6 and TNF-α) are released in response to *H. pylori* infection, which heightens the systemic inflammatory response (21, 22). Extracellular substances secreted by *H. pylori* simultaneously induce localized and chronic systemic inflammation in the mucosal layer of the stomach (23, 24). Infection with *H. pylori* leads to an increase in the generation of reactive oxygen species (ROS) and reactive nitrogen species (RNS), causing oxidative stress, which alters the thiol groups of albumin to neutralize free radicals, resulting in its own oxidative inactivation and exacerbating the development of hypoalbuminaemia (25, 26). *H. pylori* infection disrupts the integrity of the gastric mucosa, increases vascular permeability, and causes the leakage of albumin from the vasculature into the tissue interstitial space or the gut (25, 27, 28).

Our logistic regression model revealed that individuals with *H. pylori* infection had a 14.1% increased risk of MASLD (OR = 1.141, 95% CI: 1.072–1.215, *p* < 0.001), with no significant differences noted among the subgroups categorized by sex, age, smoking status, or drinking status (Figure 3). We hypothesized that Hp infection may exacerbate metabolic disorders and hepatic steatosis by altering the composition of the intestinal flora (dysregulation of the *phylum Thick-walled Bacteria*/Anthrobacterium) (29–31). In addition, an animal study revealed that CagA-positive *H. pylori* infection significantly upregulated hepatic genes related to lipid synthesis (PPAR*α*, fatty acid degradation pathway) and increased the release of inflammatory factors such as TNF-α and IL-6, leading to disruption of liver lipid metabolism and insulin resistance and encouraging fat accumulation in the liver (9, 32). Nevertheless, a 2-year cohort study indicated that *H. pylori*-infected individuals were not more likely to develop MASLD (33). Hence, conducting high-quality prospective cohort studies or randomized controlled trials is crucial for understanding the connection between *H. pylori* infection and MASLD.

The NAR integrates information from two important dimensions, inflammation and nutrition, and is an easily evaluated and cost-effective *haematological biomarker* (34). Bao (35) et al. suggested that the NAR is a noninvasive predictor of MASLD, and He et al. investigated the nonlinear link between the NAR and MASLD, utilizing RCS curves and threshold effects. Our analysis revealed that for every 1-unit increase in the NAR, the likelihood of developing MASLD increased by 3.443 times (OR = 3.443; 95% CI: 2.927–5.026; *p* < 0.001). The connection between the NAR and heightened inflammation supports the progression of hepatic insulin resistance and lipid storage (9, 36, 37), and an elevated NAR might also promote neutrophil-associated processes, such as the formation of NETs, causing injury to liver cells and fibrosis, which could promote the progression of MASLD to MASH (36, 38–40). In parallel, mediation analysis was conducted to elucidate the link between the NAR, *H. pylori* infection, and MASLD. The findings indicated that *H. pylori* infection had a partial mediating effect on MASLD via the NAR, accounting for 33.74% of the effect. *H. pylori* infection may increase the risk of MASLD by modulating chronic inflammation and nutrient metabolism.

Examining the association between *H. pylori* infection and other significant nongastrointestinal diseases could identify new targets for clinical treatment. These findings could serve as a foundation for screening for *H. pylori* infection in the population to better evaluate an individual’s risk of developing MAFLD, allowing for early intervention and prevention. This is the first study to indicate that the NAR could serve as a mediator between *H. pylori* infection and the risk of MASLD; specifically, whether decreasing the NAR through the elimination of *H. pylori* reduces the risk of MASLD could be studied, and new therapeutic targets for the prevention and treatment of MASLD could be identified. However, further research is needed to determine whether *H. pylori* infection can slow or reverse early fibrosis in MASLD.

There are several limitations to this study. First, as a cross-sectional study, it could identify only associations among *H. pylori* infection, the NAR, and MASLD, with a limited ability to prove a causal link. Furthermore, even though several potential confounders were considered, other factors might have altered the results, affecting the reliability of the study’s findings. Finally, the study sample originated from a specific region and a specific population, and there may have been some selection bias limiting the generalizability of the results, which need to be validated in a broader population in the future.

## 5 Conclusion

To summarize, the findings imply that *H. pylori* infection may indirectly increase the risk of MASLD through its impact on the NAR. Using the NAR in addition to *H. pylori* infection status enhances the accuracy of predicting MASLD risk. In addition, these results may offer a foundation for screening for *H. pylori* infection in populations to prevent MASLD and support the adoption of clinical practices aimed at changing *H. pylori* infection status. Additionally, they endorse preventive measures to decrease the rate of *H. pylori* infection, such as advancing public health efforts.

## Data Availability

The raw data supporting the conclusions of this article will be made available by the authors, without undue reservation.

## Supplementary material

SUPPLEMENTARY FIGURE S1 Unadjusted model of nonlinear connection between neutrophil-albumin ratio (NAR) with MASLD.

SUPPLEMENTARY FIGURE S2 Partially adjusted model of nonlinear connection between neutrophil-albumin ratio (NAR) with MASLD. Adjusted for age and sex.
