# Supplementary figures and images for: Helicobacter pylori infection and its impact on metabolic dysfunction-associated steatotic liver disease: a mediation analysis of neutrophil-albumin ratio
Source: Front Nutr. 2025 Dec 11;12:1701544. doi: 10.3389/fnut.2025.1701544 (PMC12739950; doi:10.3389/fnut.2025.1701544)

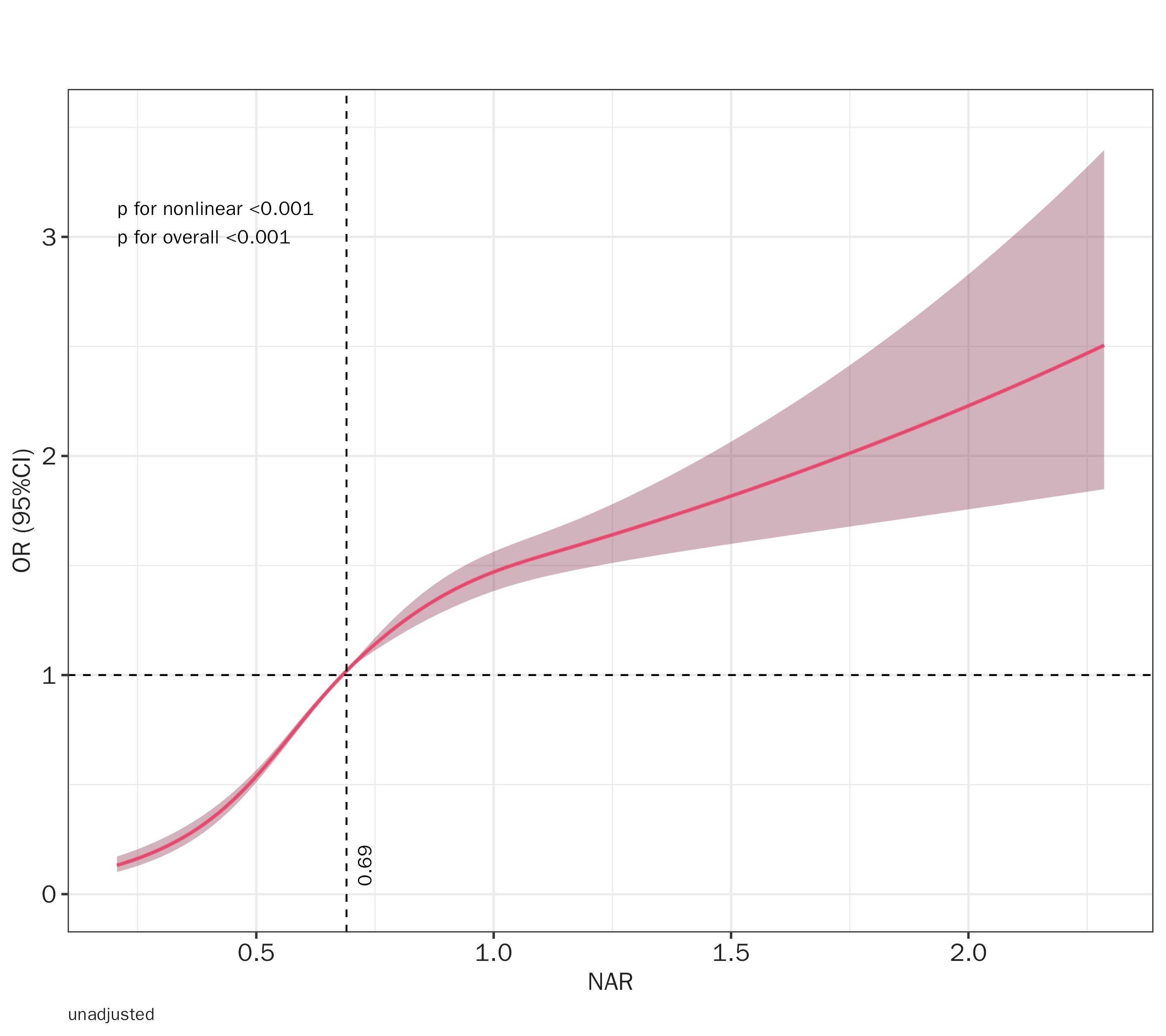

Supplement: Supplementary file 1 [file Image_1.tiff]

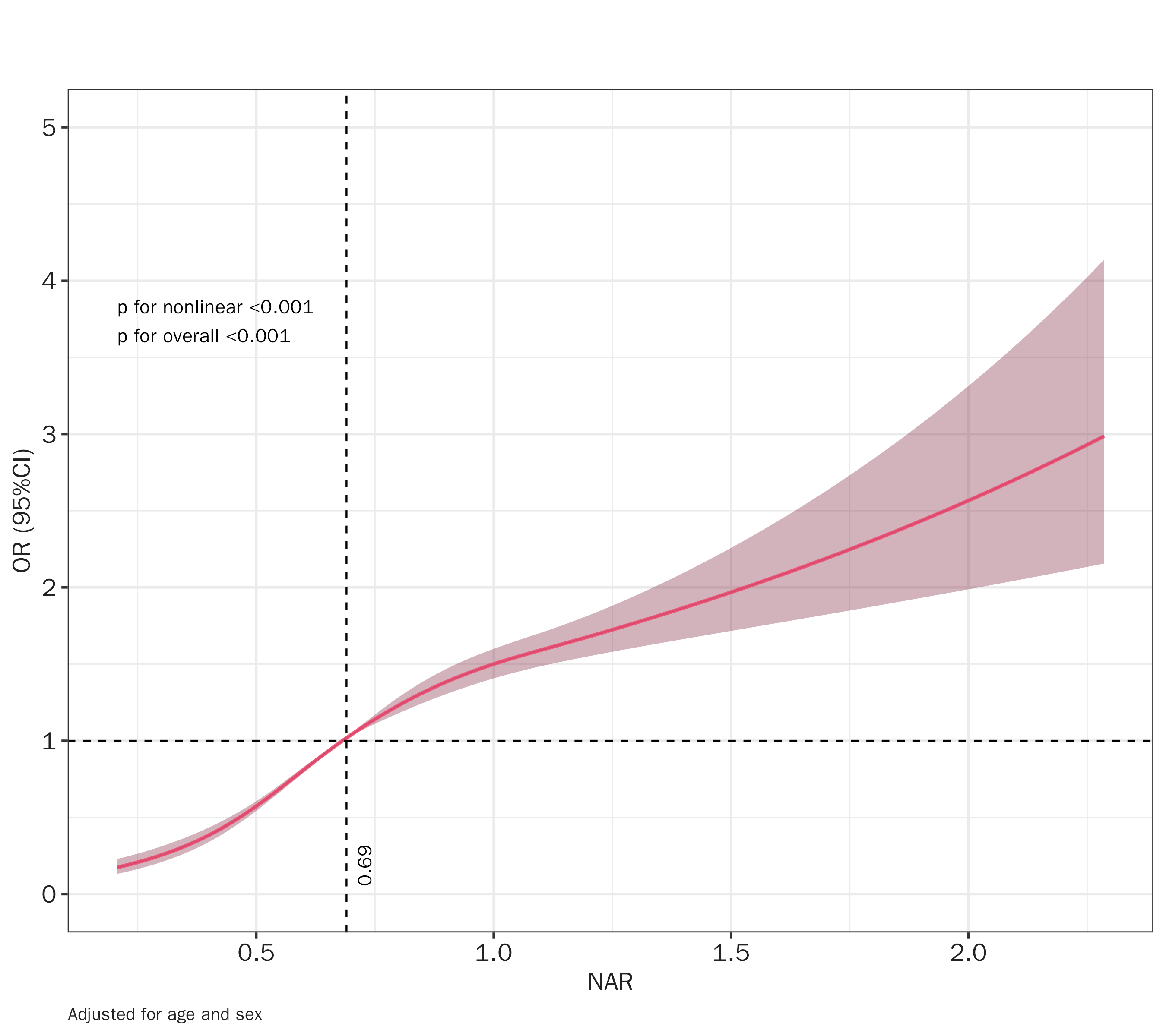

Supplement: Supplementary file 2 [file Image_2.tiff]
